# Functional outcomes of one-, two- and three-lobe technique in anatomical endoscopic enucleation for benign prostatic obstruction: a systematic review and network-meta-analysis

**DOI:** 10.1007/s00345-025-06085-3

**Published:** 2025-12-27

**Authors:** Oswald David, Sieberer Manuela, Ramesmayer Christian, Deininger Susanne, Pallauf Maximilian, Enikeev Dmitry, Herrmann Thomas, Sountoulides Petros, Lusuardi Lukas

**Affiliations:** 1 Department of Urology and Andrology, University Hospital Salzburg, Landeskrankenhaus Salzburg, Paracelsus Medical University, Muellner Hauptstrasse 48, 5020 Salzburg, Austria; 2 Rabin Medical Center (Belenson, Hasharon), Petah Tikva, Israel; 3 Faculty of Medicine, Tel Aviv University, Tel Aviv, Israel; 4 Vienna Medical University, Vienna, Austria; 5 Institute for Urology and Reproductive Health, Moscow, Russia; 6 Department of Urology, Kantonspital Frauenfeld, Spital Thurgau AG, Frauenfeld, Switzerland; 7 1st Department of Urology, Aristotle University of Thessaloniki, Thessaloniki, Greece

**Keywords:** Benign prostate hyperplasia, Prostate enucleation, Lobe, En-bloc, BipolEP, HoLEP, ThuLEP

## Abstract

**Introduction:**

Benign prostatic obstruction (BPO) is a common condition. Anatomical endoscopic enucleation of the prostate (EEP) is the mainstay in therapy for large glands (> 80 ml), but also feasible in smaller prostates. The original three-lobe enucleation technique has been supplemented by two-lobe and one-lobe or en-bloc techniques. One- and two-lobe techniques seem more effective in terms of surgical time and blood loss. The impact of the different techniques on functional outcomes however is unclear.

**Objective:**

To determine whether the technique used in prostate enucleation influences functional outcomes of surgery.

**Methods:**

We performed a systematic review of Medline/Pubmed and identified 124 studies, of which 10 were included for network meta-analyses. We included RCTs as well as retrospective and prospective cohort series, comparing one-lobe, two-lobe and three-lobe technique respectively. We assessed functional outcomes including post-void residual urine, Qmax (ml/s), IPSS Score and QoL Score. Additionally we compared stress and urge incontinence rates after three months.

**Results:**

The only significantly differing result was in postoperative Qmax increase in favor of one-lobe and two-lobe compared to three-lobe technique. However, with mean differences of 0.989 and 0.749 respectively the effect was clinically negligible. There was no difference in the other functional outcomes including incontinence rates.

**Limitations:**

Our approach is limited by the quality of the data, since retrospective and non-randomized trials were included due to the overall poor amount of studies on the subject.

**Conclusion:**

One-lobe/en-bloc, two-lobe and three-lobe technique in endoscopic enucleation of the prostate for benign prostate syndrome all improve micturition. No final conclusion concerning superiority of a specific lobe-technique in terms of functional outcomes can be drawn from the available data. Randomized controlled trials are needed.

## Introduction

Benign prostate enlargement (BPE) with consecutive lower urinary tract symptoms (LUTS) is a common disease with an age-standardized prevalence of 2.480/100.000 and increasing disease burden due to aging societies [1].

After failure of behavioral and medical therapy a variety of minimally invasive, endoscopic and open surgical procedures are available for treatment [2, 3]. Traditionally open simple prostatectomy was used for large glands (> 80 ml), but has widely been superseded by endoscopic enucleation of the prostate (EEP), which is considered the gold-standard by the European Association of Urology (EAU) for these patients [2]. EEP has shown decreased morbidity especially in terms of blood loss, transfusion rates, catheterization time and length of hospital stay [4]. Subsequently EEP was also shown to be feasible and profitable in smaller glands with an equally low complication rates as traditional transurethral resection of the prostate (TUR-P) [5, 6]. The EAU recommends EEP as alternative procedure to TUR-P in glands between 30-80ml [2].

The concept of endoscopic enucleation of the prostate mimics the one of open simple prostatectomy, namely the complete removal of the adenoma with preservation of the prostatic capsule. After enucleation of the prostate a morcellation device is customarily used to shred the prostatic lobes in order to remove them from the bladder and prostatic fossa [7].

A variety of different energy sources such as monopolar, bipolar (BipolEP), Holmium laser (HoLEP) and Thulium laser (ThuLEP) have been described. All of them seem equally feasible and effective, with a reported advantage of laser techniques in control of bleeding and electrical current in terms of overall surgery time [8]. Similar learning curves with adequate quality of enucleation after 20–30 cases of supervised training have been proposed [9, 10]. Overall quality of EEP seems to be less dependent on energy source than technique [7, 11, 12].

In terms of surgical procedure traditionally a three-lobe technique with incision and separate enucleation of middle and lateral lobes has been described [13–15]. However, two-lobe techniques with joint resection of median and one lateral lobe and en-bloc or one-lobe techniques [16–20] are well established in clinical practice [21].

All techniques are well described and showed adequate intraoperative and functional outcomes, however there is a lack of comparative data on different surgical techniques. A meta-analysis by Ortner et al. from February 2023 investigated 6 randomized controlled trials (RCT) comparing different approaches in EEP including power settings, pulse modulations and also lobe technique [22]: Yet only two RCTs comparing different resection approaches could be identified. Overall one- and two lobe technique resulted in reduced operative time and less blood loss compared to three-lobe technique, however no differences could be shown in terms of functional results.

In order to update and further investigate the influence of different lobe techniques on functional outcomes of EEP, we conducted a systematic review and network meta-analysis comprising all RCTs and controlled case-series on the matter.

## Material and methods

### Eligibility criteria

Inclusion criteria: We included controlled observational trials, controlled retrospective trials and RCTs in English language. In terms of PICO criteria studies must report on functional outcomes of different resection techniques, in terms of one-, two- or three-lobe resection, in anatomic prostate enucleation for benign prostatic syndrome. We included RCTs as well as cohort series. Data cutoff was 31st of October 2024.

Exclusion Criteria: Articles in other languages than English, editorials, letters to the editor, case reports were excluded from the study. Also, single-arm studies were excluded from systematic review.

### Information sources

Pubmed/Medline was searched systematically. Reference lists of screened publications were also screened for additional publications, which could not be identified by the pre-specified keywords.

### Search strategy:

The pre-specified keywords were used to search the pre-specified database in different combinations, including a synonym for prostate enucleation and resection technique.

Keywords: ((prostate enucleation) OR (enucleation of the prostate) OR (EEP)) OR (HoLEP)) OR (ThuLEP)) OR (BipolEP)) AND ((en bloc) OR (one lobe) OR (two lobe) OR (three lobe)).

### Study records

Data management: All identified studies were recorded using Mendeley [23]. Data retrieved during data extraction phase was saved using Microsoft Excel [24] spread sheets.

**Fig. 1:**
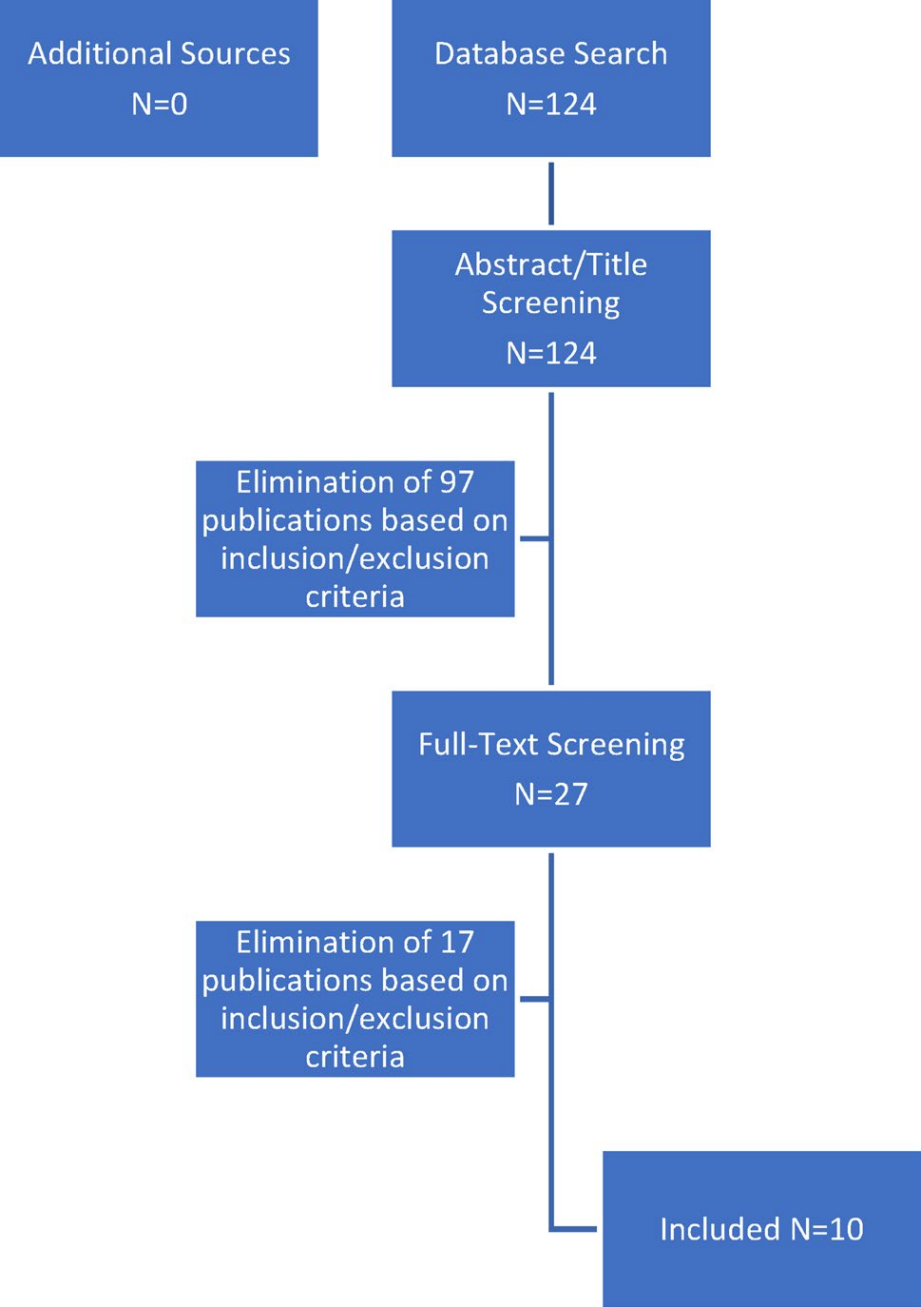
Flowchart of study selection process

Selection Process: Two independent reviewers screened the databases according to the keywords for studies, excluded duplicates and checked for eligibility by review of title and abstract. Full text read was performed for unclear cases. The selection of both authors was then compared for discrepancies. In such cases, a third reviewer was asked to aid in decision-making. A detailed flowchart of the selection process is depicted in Fig. 1.

Data Collection Process: Data was extracted according to a pre-specified Excel sheet by both authors to avoid typing errors.

### Data items

Baseline parameters: Pre-specified baseline data included patient age, preoperative PSA value (ng/ml), prostate volume (cc), preoperative post-void residual urine (PVR) (ml), preoperative QMax (ml/s), preoperative International Prostate Symptom Score (IPSS), preoperative Quality of Life Score (QoL) and the presence of a catheter preoperatively.

Study parameters: postoperative PVR (ml), QMax (ml/s), IPSS Score, QoL Score were recorded. If presented as change in PVR, QMax, IPSS or QoL score in respect to preoperative values the data was recorded as well. Furthermore we looked at stress and urge urinary incontinence outcomes three month postoperatively.

### Risk of bias assessment

Risk of bias assessment was performed using the Robins-1 tool [25] for non-randomized studies. Additionally we used the Cochrane RoB 2 tool [26] for RCTs. Since there, obviously, was no investigator blinding in the two included RCTs [27, 28], they had to be classified as “high risk of bias”. We are aware of the overall high risk of bias of the included studies, but we thought this justified in light of the scarce data on the matter.

### Data synthesis

A frequentist network meta-analysis was created to compare all operation techniques. All pairwise direct and indirect comparisons between all operations methods are presented using appropriate effect sizes (for continuous variables the mean differences and for dichotomous variables odds ratios were used) and two-sided 95% confidence intervals. Based on the estimated heterogeneity between the studies, which was assessed using Higgins I^2^ and Cochran’s Q, a common effects or random effects model was used. Operation methods were ranked based on P-Scores, which measure the mean extent of certainty that a treatment is better than the competing operation methods (0 means theoretically worst and 1 means best). Funnel plots and Egger’s test for asymmetry were used to assess potential publication bias.

If no mean and standard deviation were given, but median and interquartile range, Wan’s approach was used to estimate mean and standard deviation [29].

A p-value < 0.05 was taken as the uncorrected statistical significance level (two-sided); therefore, all inferential results are only descriptive. For statistical analysis, the statistical computing software R, Version 4.4.3 (R Foundation for Statistical Computing, Vienna, Austria. URL http://www.R-project.org), was used. For conducting the network meta-analysis, the R package netmeta was used [30].

## Results

10 studies were included in the network meta-analysis, which contained 8, 7 and 8 cohorts of one-lobe, two-lobe and three lobe EEP respectively. There was no study on BipolEP, two studies on ThuLEP, one study on ThuLEP and HoLEP and seven studies on HoLEP only. (Table 1).

**Table 1:** Included Studies

Author	Study Type	Timeline	Patient number (1/2/3 lobe)	Energy	Risk of Bias
Saredi et al.	Prospective consecutive series	2015–2016	n = 100 (50/0/50)	ThuLEP	Serious (ROBINS-I)
Enikeev et al.	Retrospective consecutive series	2013–2018	n = 1115 (406/709/0)	HoLEP, ThuLEP	Serious (ROBINS-I)
Xu et al.	RCT	2014–2017	n = 191 (0/97/94)	HoLEP	High (RoB 2)
Tokatli et al.	Retrospective consecutive series	2015–2017	n = 178 (59/60/59)	HoLEP	Serious (ROBINS-I)
Liu et al.	Cons. prospective series, cons. retrospective controls	2016–2019	n = 120 (60/0/60)	HoLEP	Serious (ROBINS-I)
Rücker et al.	RCT	2017	n = 600 (200/200/200)	HoLEP	High (RoB 2)
Tuccio et al.	Retrospective consecutive series	2017–2019	n = 168 (87/0/81)	HoLEP	Moderate (ROBINS-I)
Tamalunas et al.	retrospective, propensity score-matched analysis	2017–2020	n = 606 (303/0/303)	HoLEP	Low (ROBINS-I)
Mahajan et al.	RCT	2020–2021	n = 64 (30/34/0)	HoLEP	Moderate (ROBINS-I)
Cantiello et al.	retrospective, propensity score-matched analysis	2019–2024	n = 213 (71/71/71)	ThuLEP	Low (ROBINS-I)

### Baseline parameters

The included studies comprised comparable patient populations in terms of age, prostate volume and PSA value among the surgical technique groups. Baseline functional parameters, such as PVR, Qmax, IPSS Score, QoL Score and the presence of a catheter were also balanced. A descriptive presentation is given in Table 2.

**Table 2:** Descriptive depiction of baseline parameters

	One-lobe	Two-lobe	Three-lobe
Age			
n_total_	1206	1231	918
n_average_	151	176	115
# of studies	8	7	8
Weighted mean	69.1	68.1	70.4
Weighted SD	8.4	7.8	7.7
PSA preOP (ng/ml)			
n_total_	1206	1231	918
n_average_	151	176	115
# of studies	8	7	8
Weighted mean	5.1	4.7	5.5
Weighted SD	4.4	3.8	4.2
Prostate Vol. (CC)			
n_total_	1206	1231	918
n_average_	151	176	115
# of studies	8	7	8
Weighted mean	90.2	87.3	89.9
Weighted SD	42.6	40.6	42.1
PVR preOP (ml)			
n_total_	1176	1197	868
n_average_	168	200	124
# of studies	7	6	7
Weighted mean	105.9	100.8	143.6
Weighted SD	78.4	76.9	117.3
Qmax preOP (ml/s)			
n_total_	1156	1231	868
n_average_	165	176	124
# of studies	7	7	7
Weighted mean	9.3	9.0	8.9
Weighted SD	3.5	3.7	4.3
IPSS preOP			
n_total_	1206	1231	918
n_average_	151	176	115
# of studies	8	7	8
Weighted mean	21.5	21.6	21.0
Weighted SD	5.2	4.7	6.6
QoL preOP			
n_total_	1097	1171	809
n_average_	183	195	135
# of studies	6	6	6
Weighted mean	4.2	4.2	4.3
weighted SD	1.8	1.0	1.2

### Postoperative PVR (ml)

**Fig. 2:**
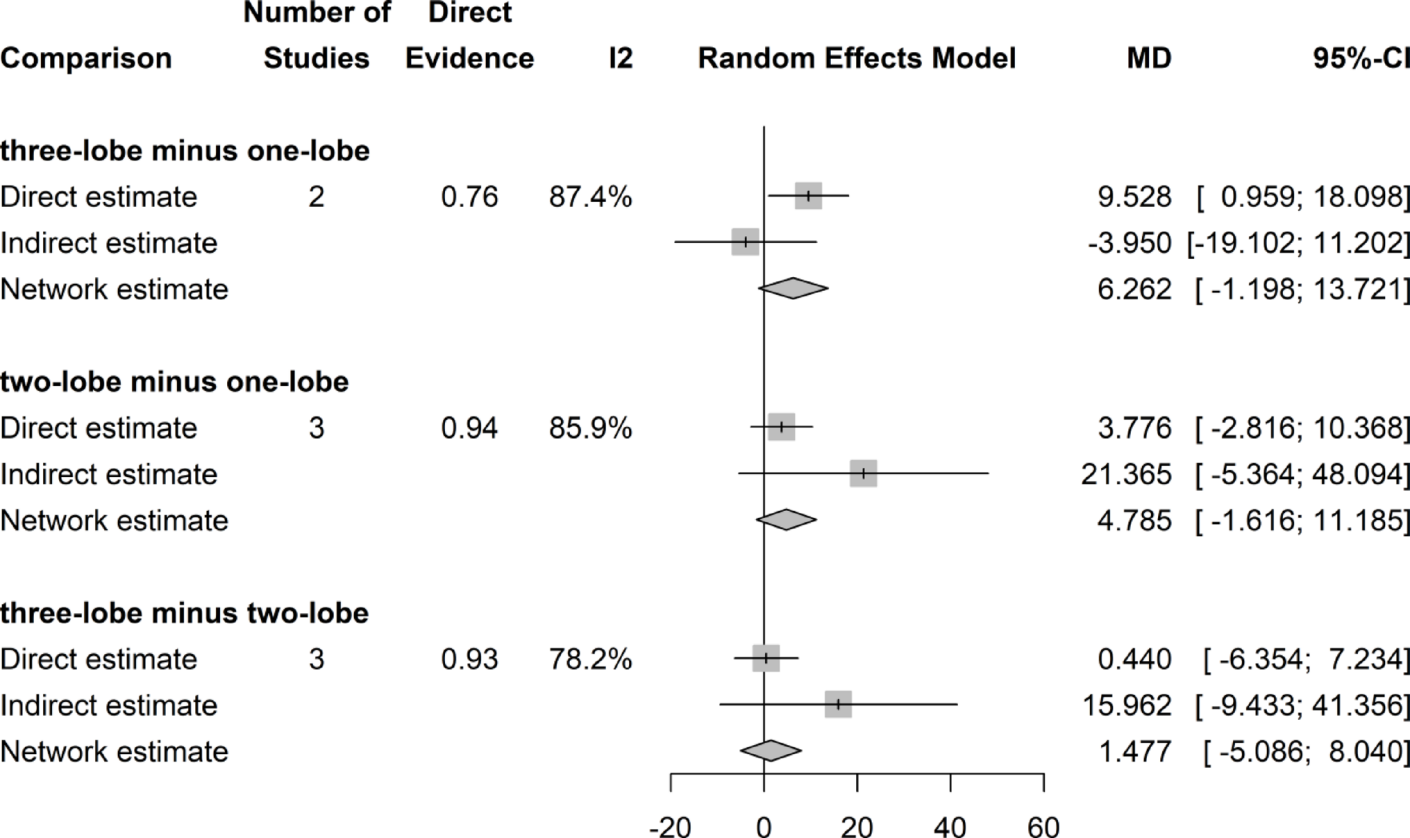
Forrest plot: PVR network meta-analysis

There was no significant difference in PVR with regard to the surgical technique (Fig. 2).

### PVR change (ml)

**Fig. 3:**
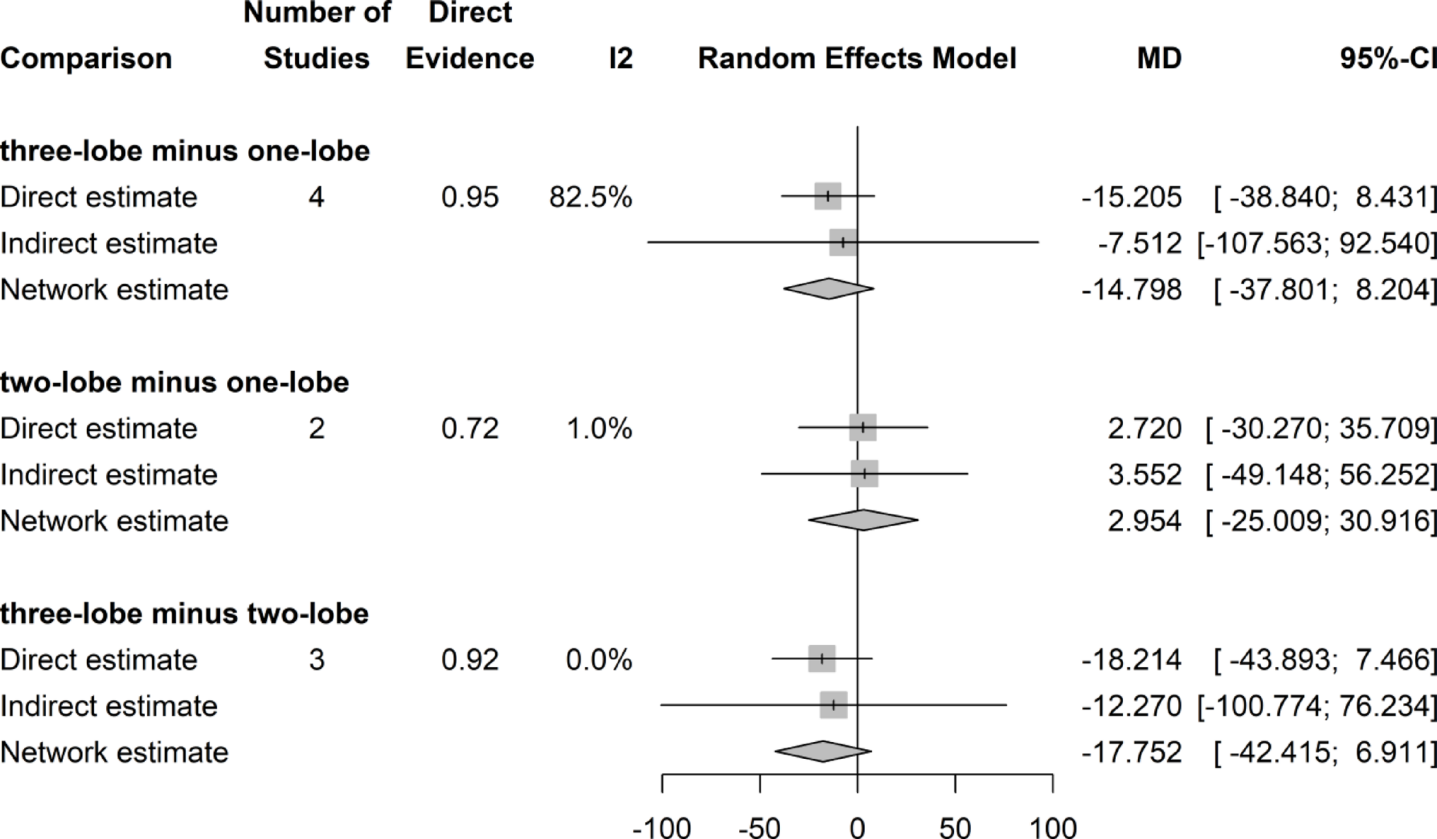
Forrest plot: Change (decrease) in PVR after surgery network meta-analysis

There was no significant difference in PVR decrease with respect to the preoperative value throughout (among) the different surgical techniques (Fig. 3). For this analysis the Funnel plot showed asymmetry of results and the Egger’s test was positive (p = 0.039), both of which indicate a possible publication bias.

### Postoperative Qmax (ml/s)

**Fig. 4:**
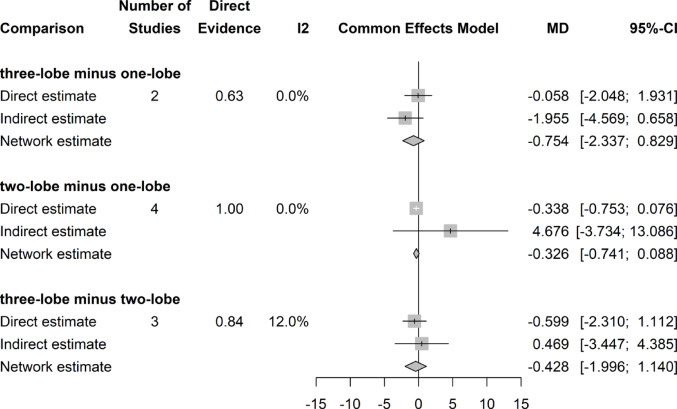
Forrest plot: Qmax network meta-analysis

There was no significant difference in postoperative flow studies, presented by means of Qmax throughout all three surgical techniques (Fig. 4).

### Qmax change (ml/s)

**Fig. 5:**
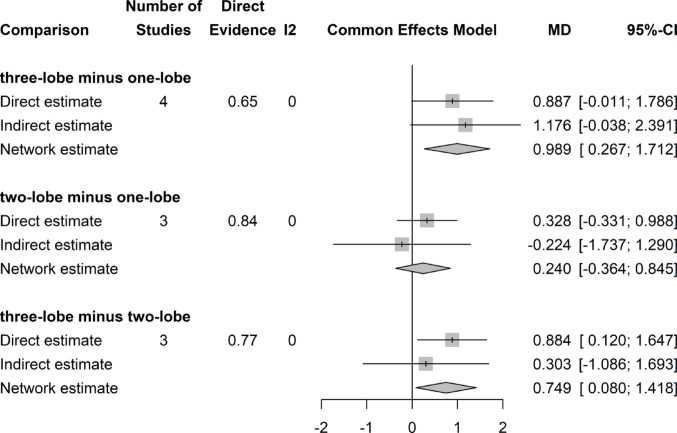
Forrest plot: Change (Increase) in Qmax after surgery network meta-analysis

Network meta-analysis showed a marginal, but significant difference in postoperative Qmax increase with respect to preoperative values for two-lobe (MD 0.749; 95%CI 0.080, 1,418) and one-lobe (MD 0.989; 95%CI 0.267, 1,712) techniques compared with the three-lobe technique. There was no difference between two- and one-lobe technique (Fig. 5).

## Postoperative IPSS score

**Fig. 6:**
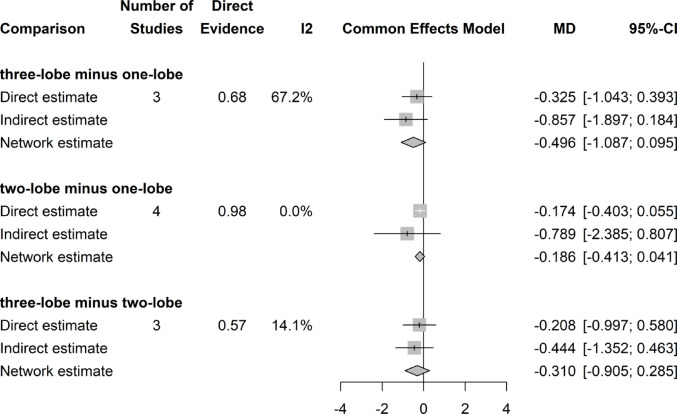
Forrest plot: IPSS network meta-analysis

There was no significant difference in postoperative IPSS Scores throughout the three groups (Fig. 6).

### IPSS score change

**Fig. 7:**
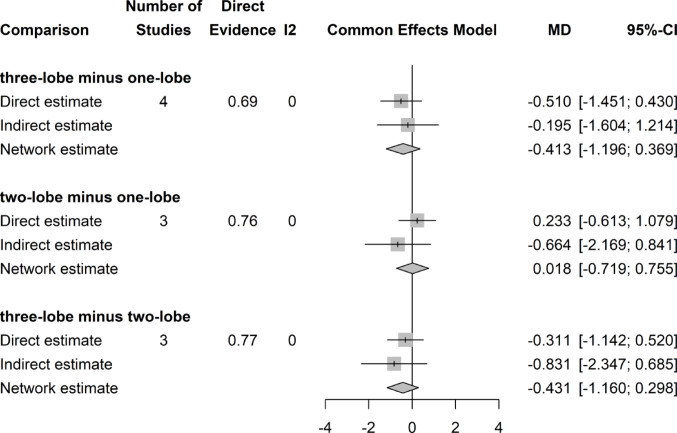
Forrest plot: Change (decrease) of IPSS Score after surgery network meta-analysis

There was no significant difference in IPSS Score decrease after surgery throughout all three surgical groups (Fig. 7).

### Postoperative QoL score

**Fig. 8:**
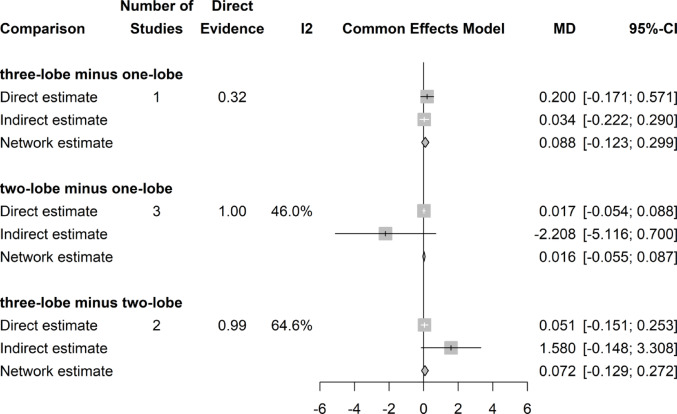
Forrest plot: QoL Score network meta-analysis

There was no significant difference in postoperative QoL Scores among the three groups (Fig. 8).

### QoL score change

**Fig. 9:**
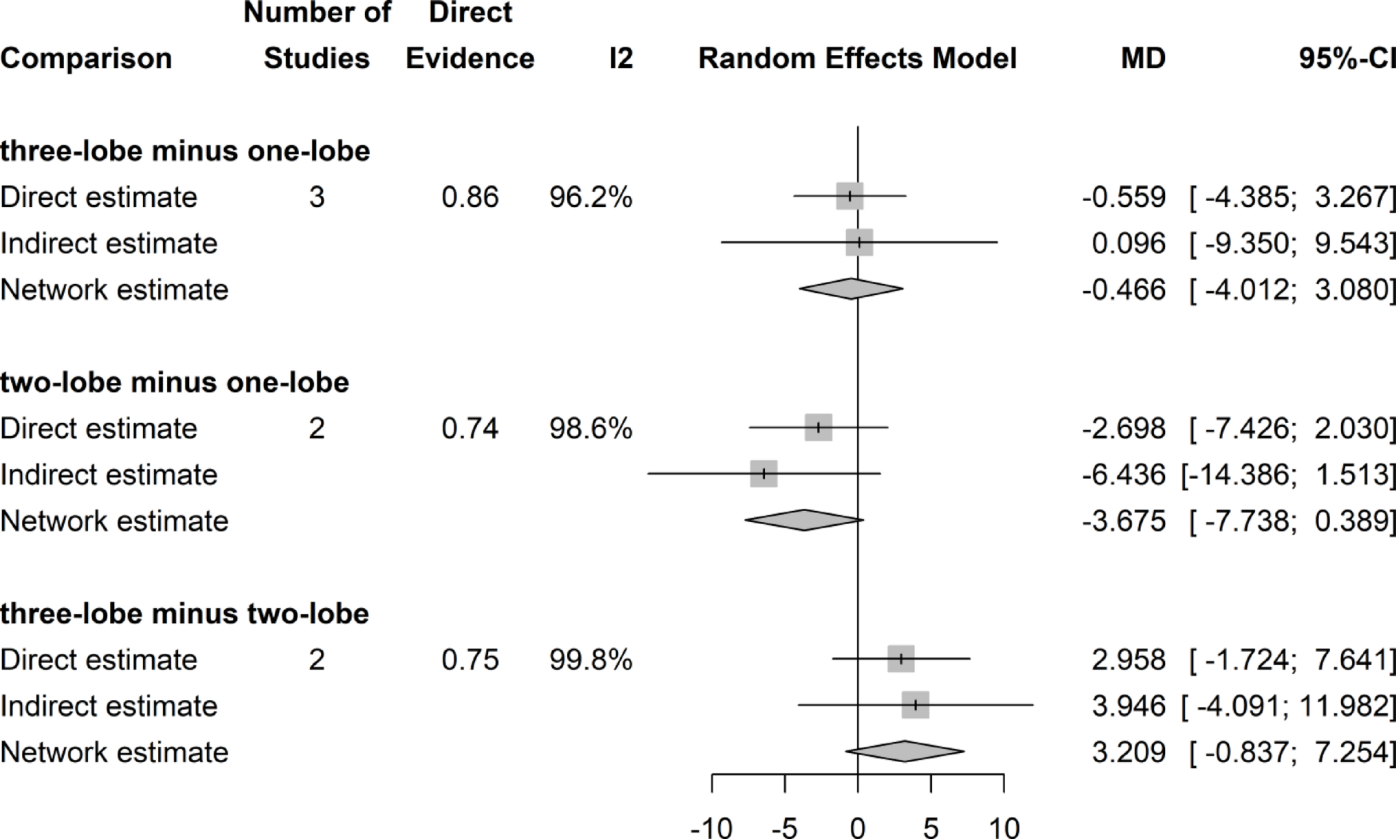
Forrest plot. Change of QoL score (decrease) after surgery network meta-analysis

There was no significant difference in QoL Score decrease after surgery among all three surgical groups (Fig. 9).

### Postoperative urinary stress incontinence

**Fig. 10:**
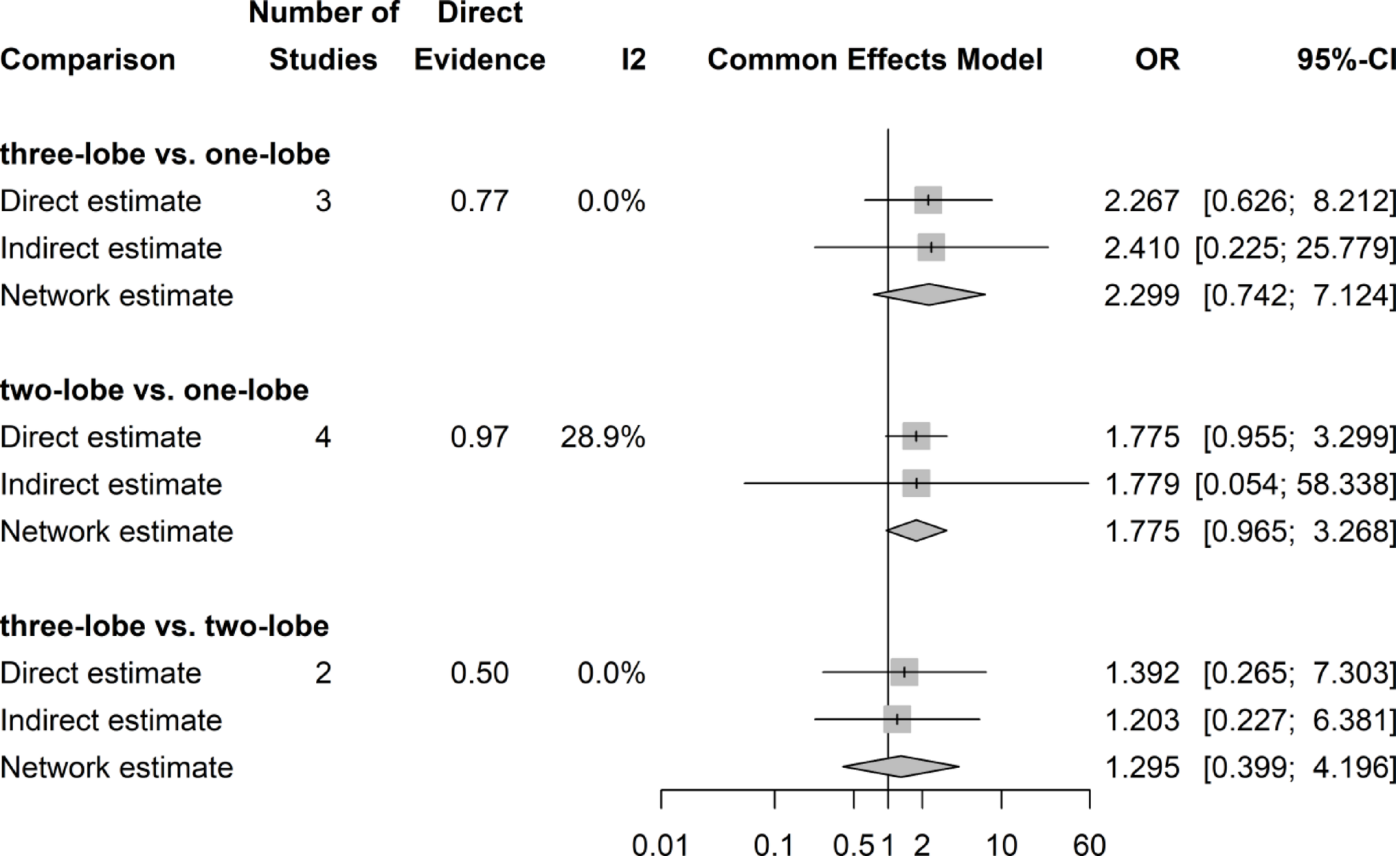
Forrest plot: Postoperative urinary stress incontinence (3 months) network meta-analysis

No significant difference in the rate of stress urinary incontinence at three months postoperatively could be shown regardless of surgical approach (Fig. 10).

### Postoperative urinary urge incontinence

**Fig. 11:**
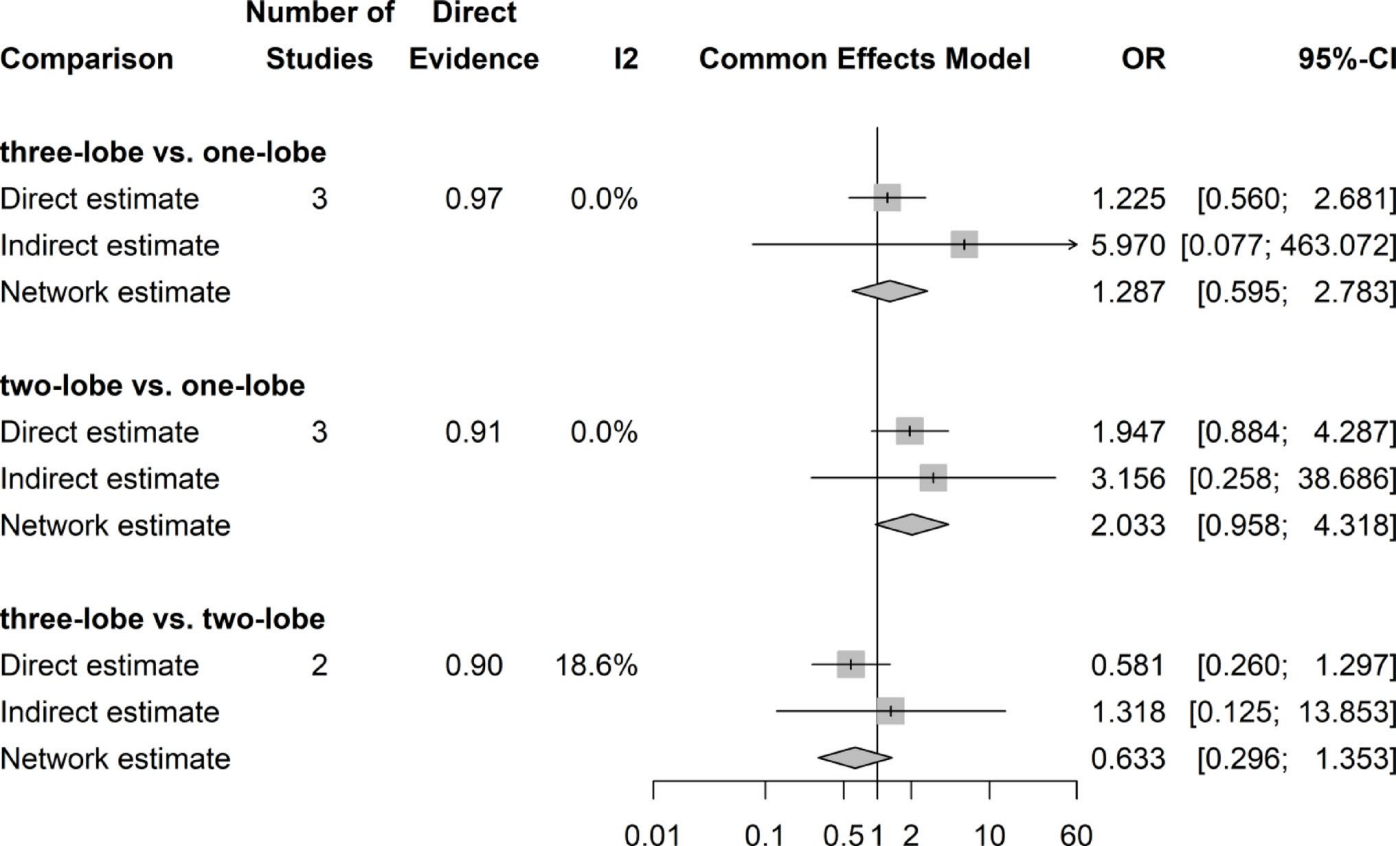
Forrest plot: Postoperative urinary urge incontinence (3 months) after surgery network meta-analysis

No significant difference in urge urinary incontinence rates were shown in our analyses (Fig. 11). There was a slight asymmetry of results on Funnel plot, but the Egger’s test was negative.

## Discussion

In an effort to evaluate the impact of the different techniques of anatomical EEP on functional outcomes we assessed ten trials, comprising of eight, seven and eight surgical cohorts investigating one-lobe/en-bloc, two-lobe and three- lobe anatomical EEP technique respectively. These included three RCTs and two retrospective propensity-score matched analyses. The three groups comprised of 1206, 1231 and 918 patients respectively. Our work represents an update and supplement to a meta-analysis by Ortner et al. They investigated multiple aspects of technique in anatomical EEP, including two RCTs on lobe-technique [22].

We determined age, prostate volume and PSA value as most important baseline parameters, all of which were balanced among the three study groups. Age and prostate volume were found to be risk factors for transient postoperative incontinence after HoLEP/BipolEP [31, 32].

Preoperative functional parameters measured were PVR, Qmax, IPSS Score and QoL Score. Except for slightly higher residual urine volumes in the three-lobe group, all of them seemed well balanced. Roughly a third of patients throughout all groups had an indwelling catheter at the time of surgery, however most trials did not report on this issue. Only two trials included data on use of anticoagulation drugs, which was the case for around 60% in all groups. The type of anticoagulation was presented too divergent as to allow a valid comparison.

**Fig. 12:**
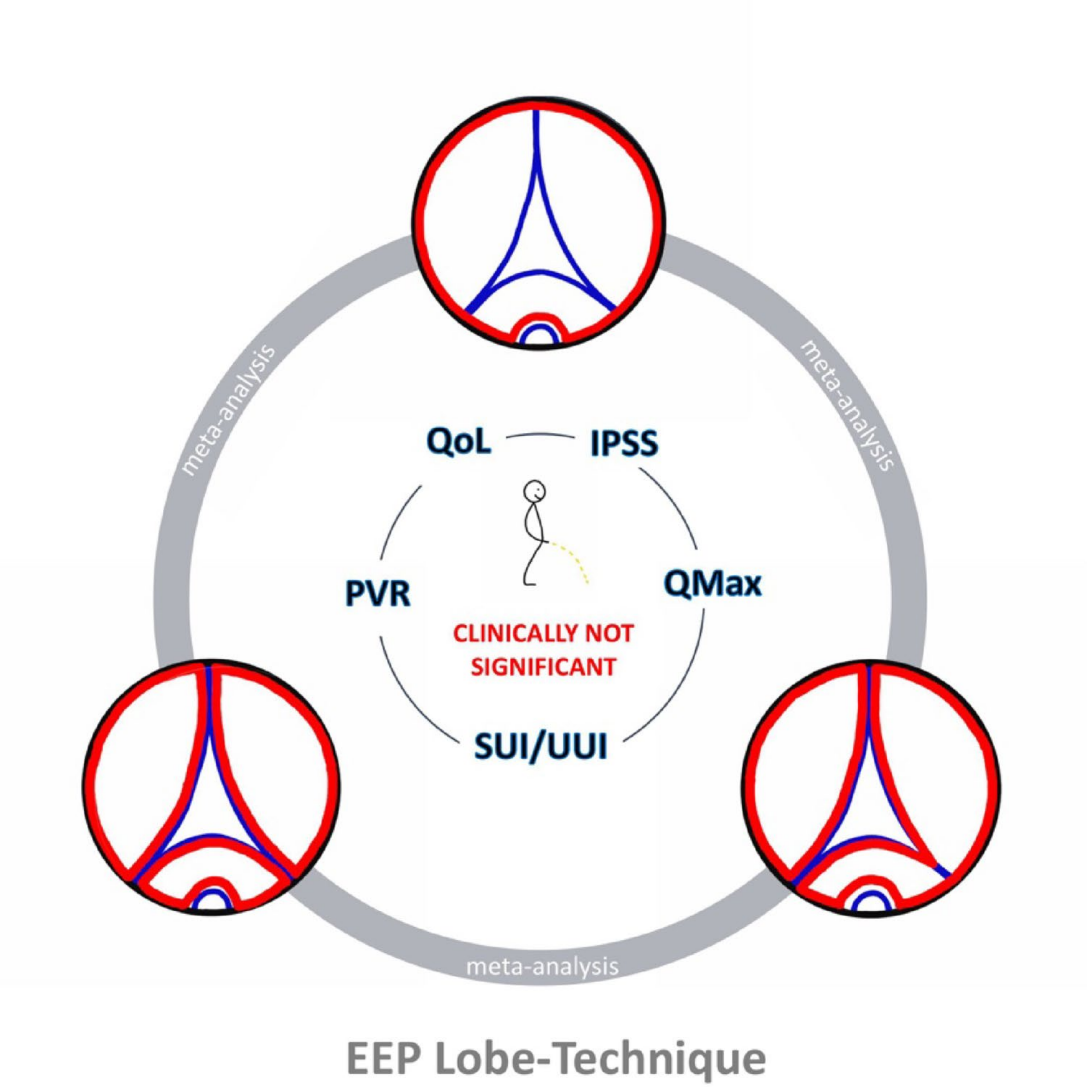
Graphical depiction of findings

We conducted a network meta-analysis comparing one-lobe/en-bloc, two-lobe and three lobe enucleation techniques in respect of their functional outcomes. We considered postoperative PVR, Qmax, IPSS Score, QoL Score and furthermore reduction in PVR, increase in Qmax, reduction in IPSS score and QoL Score. A graphical depiction of our results can be found in Fig. 12.

The only significantly differing result we found was in postoperative Qmax increase in favor of one-lobe and two-lobe compared to three-lobe technique. However with mean differences of 0.989 and 0.749 respectively this effect has to be considered clinically negligible. All other analyses including postoperative Qmax were negative regardless of the surgical technique.

Additionally we looked at the rate of SUI and UUI at three months postoperatively. Five and four studies presented data on the parameters and could be used for analysis. The time of analysis was chosen because most studies presented three-month data on the topic. Furthermore there is a known expected period of transient incontinence postoperatively in EEP, which is usually resolved within the first three months [32, 33]. We specifically wanted to assess incontinence rates and severity as a surrogate of lobe-technique quality. There was no significant difference in SUI or UUI comparing all three groups. In general numbers of patients with reported incontinence were low with varying percentages depending on the sample size, ranging from 0 to 8% for SUI/UU.

A variety of confounders has to be discussed, which might have had similar or even greater impact on surgical results than mere lobe-technique.

A substantial number of BPS patients show some amount of incontinence preoperatively, which is not assessed in most studies [34]. Also most studies do not report on incontinence in a standardized fashion, post-operative time points of evaluation varied and de-novo is not distinguished from preexisting incontinence. Thus postoperative SUI/UUI as a surrogate marker for enucleation quality has to be interpreted with caution.

Surgeon experience has been shown to correlate with postoperative incontinence rates [35] with an experience of > 40 procedures being a predictive factor for reduced urinary incontinence rates after HoLEP. This fact highlights the need for mentoring programs during the early learning curve of EEP [9]. Also commonly surgeons learning EEP start with “more-lobe” techniques before moving to en-bloc/one-lobe enucleations, which is badly represented in the literature. An influence of this learning-curve on functional outcomes can thus be assumed outside RCTs with study protocols specifically taking into account surgeons’ experience.

In terms of surgical technique, the so called “early apical release” helps to divide the urethral mucosa from the external sphincter as early in the procedure as possible. Thus traction on the sphincter fibres is reduced to a minimum [36]. Most often this technique is specified for one-lobe/en-bloc approaches, however most three- and also two-lobe approaches with an antegrade enucleation direction imply an early release.

Conversely en-bloc anterior–posterior dissection has been shown to be a risk factor for postoperative transient incontinence in HoLEP [32].

A variety of other techniques and technique modifications have been reported: Scoffone et al. described the “En-bloc no touch” technique for HoLEP with decreased tension to the capsule [20]. Ito et al. suggest a new “complete en-bloc” technique and performed a three-armed trial to compare with three-lobe and conventional en-bloc HoLEP. They reported no difference in functional outcomes, but significantly faster enucleation [18].

Furthermore different Laser Settings [37, 38] and Energy Pulse Shapes [39, 40] can influence outcomes in EEP. However there does not seem to be an influence of energy source on functional otucomes [8]. Current data suggest a difference in terms of surgical efficiency of different lobe techniques: en-bloc and two-lobe techniques seem to be beneficial compared to three-lobe techniques considering operative time and energy use [41]. No difference was shown for en-bloc versus two-lobe technique [19].

The major limitation of our work is the quality of the available data with respect to design, methodology and type of studies that were included in our meta-analysis. Due to the scarce amount of data on the matter we decided to include not just RCTs, but all available comparative trials including retrospective and prospective case series. By doing so we accepted a higher risk of bias in our analysis including all of the discussed confounders.

Some trials assessed surgical techniques sequentially and thus at different level of experience of the surgeons, which might have distorted some results.

There were differences in the surgical technique within groups as many authors use their own modification of a one-, two- or three-lobe approach with distinct surgical steps, some of which might have had a different effect on the results than the lobe-technique itself.

Also underreporting and a lack of standardization in some parameters, especially concerning incontinence, made it hard to interpret some findings.

Still we were able to comprise and analyse data of 10 different trials with more than 900 cases in each study arm, which gives an insight in the role of lobe-technique in EEP with respect to functional outcomes.

So far, no conclusive evidence of either technique’s influence on functional results has been shown. Our network meta-analyses support this notion and suggest that all of them can be equally beneficial for patients treated with anatomical EEP for obstructive LUTS due to BPE. Other aspects of surgical technique, like performance of an early apical release, and surgeon experience seem to be more important factors regarding the matter. Also underreported preoperative parameters like the presence of incontinence might influence functional outcomes. Thus no final conclusion can be drawn on superiority of any lobe-technique over the other. More (multicentric) randomized controlled trials with appropriate stratification to these confounders, detailed protocols and standardized evaluation of outcomes are warranted to draw final conclusions.

## Conclusion

One-lobe/en bloc, two-lobe and three-lobe technique in EEP effectively improve micturition in men with BPS. Due to limited data no final conclusion can be drawn concerning the superiority of either technique. RCTs are needed.

## Data Availability

The research data can be requested from and accessed by the corresponding author.

## Abbreviations

BPE: Benign prostate enlargement
EEP: Endoscopic Enucleation of the Prostate
LUTS: Lower urinary tract symptoms
EAU: European Association of Urology
TUR-P: Transurethral resection of the prostate
BipolEP: Bipolar Enucleation of the Prostate
HoLEP: Holmium laser Enucleation of the Prostate
ThuLEP: Thulium laser Enucleation of the Prostate
RCT: Randomized Controlled Trial
PVR: Post-void residual urine
IPSS: International Prostate Symptom Score
QoL: Quality of Life (Score)
